# Combined Effect of *Nigella sativa* and Kefir on the Live Performance and Health of Broiler Chickens Affected by Necrotic Enteritis

**DOI:** 10.3390/ani14142074

**Published:** 2024-07-15

**Authors:** Vishal Manjunatha, Julian E. Nixon, Greg F. Mathis, Brett S. Lumpkins, Zeynep B. Güzel-Seydim, Atif C. Seydim, Annel K. Greene, Xiuping Jiang

**Affiliations:** 1 Department of Food, Nutrition and Packaging Sciences, Clemson University, Clemson, SC 29631, USA; vmanjun@clemson.edu; 2 Department of Animal and Veterinary Sciences, Clemson University, Clemson, SC 29631, USA; jenixon@clemson.edu (J.E.N.); southern_poultry_res@msn.com (G.F.M.); southernpoultry@gmail.com (B.S.L.); or zeynepseydim@sdu.edu.tr (Z.B.G.-S.); or atifseydim@sdu.edu.tr (A.C.S.); agreene@clemson.edu (A.K.G.); 3 Southern Poultry Feed & Research, Inc., Athens, GA 30607, USA; 4 Department of Food Engineering, Süleyman Demirel University, Isparta 32260, Turkey

**Keywords:** coccidiosis, necrotic enteritis, live performance, *Nigella sativa* (black cumin), kefir, broiler, *Clostridium perfringens*, cecum

## Abstract

**Simple Summary:**

Necrotic enteritis is a serious disease in chickens caused by bacterium *Clostridium perfringens*, leading to high death rates, poor growth, and an economic loss of $6 billion annually worldwide. Currently, antibiotics are commonly used to treat the disease in chickens. However, the rise in antibiotic-resistant bacteria and the growing demand for antibiotic-free poultry products highlights the need to explore sustainable alternatives. Our study investigated natural products of *Nigella sativa* (black cumin) and authentic kefir as sustainable options in reducing necrotic enteritis in chickens. An animal trial was conducted in which chickens were fed 5% black cumin seeds in their feed, 20% kefir in drinking water, and a combination of both during disease. The results showed that black cumin seeds, kefir, and their combination treatments did not have a detrimental impact on the growth of the chickens and successfully reduced infection and death rates, with the combination being the most effective. Therefore, *Nigella sativa*, kefir, and their combination can be used as sustainable alternatives to antibiotics in mitigating necrotic enteritis in chickens.

**Abstract:**

Coccidiosis and necrotic enteritis (NE) are prevalent poultry ailments worldwide, leading to decreased live performance and elevated mortality rates without antibiotic usage. This study evaluated *Nigella sativa* (black cumin) seeds (BCS) and kefir as alternatives to antibiotics for broilers. An in vivo study over a 28-day period, using 384 Cobb 500 male broilers organized into six treatment groups as part of a completely randomized block experimental design was conducted. Each treatment group included eight replicates, with each replicate containing eight birds. The treatments included positive control, negative control, antibiotic control, 5% BCS in feed, 20% kefir in drinking water, and a combination of 5% BCS and 20% kefir. NE was induced in broilers by administering ~5000 oocysts of *Eimeria maxima* orally on day 14, followed by inoculation with about 10^8^ CFU/mL of *Clostridium perfringens* (Cp) (strain Cp#4) on days 19, 20, and 21. Live performance metrics including feed intake, body weight gain, and feed conversion were assessed in broilers. Additionally, NE disease outcomes such as lesion scores, mortality rates, and Cp populations in cecum were determined during the study. The BCS, kefir, and the combination had no detrimental effect on broiler live performance. BCS-treated and combination groups had lower NE scores (*p* > 0.05) in comparison to the positive control and exhibited no significant difference (*p* > 0.05) from antibiotic control. Additionally, treatment groups and antibiotic control were not significantly different (*p* > 0.05) in mortality, whereas the BCS and kefir combination significantly reduced (*p* < 0.05) mortality to 14.1% compared to 31.3% for the positive control. *C. perfringens* vegetative cells significantly decreased (*p* < 0.05) in treatments with BCS, kefir, and their combination on days 22 and 28 compared to the positive control. On day 22, Cp sores were significantly lower (*p* < 0.05) for the kefir and combination treatments compared to the positive control. In conclusion, BCS and kefir successfully reduced *C. perfringens* infection and mortality without any detrimental impact on broiler live performance with the combined treatment being the most effective. These results suggest that BCS and kefir could serve as potential alternatives to antibiotics in managing NE.

## 1. Introduction

Necrotic enteritis (NE) is an enteric disease of global importance in poultry, evidently impacting animal well-being and posing economic challenges. With causative agents being toxin types A, C, and G of the Gram-positive bacterium *Clostridium perfringens*, the disease is an enterotoxaemia and is apparent when bacterial abundance is accompanied by substantial impairment of the gastrointestinal epithelium [1]. Coccidiosis, caused by different species of the parasite *Eimeria*, that damage the gut epithelium, is a predisposing factor for NE, leading to acute and deadly disease in poultry [2].

Necrotic enteritis is frequently induced post-coccidial infection or by factors which cause stress, diminishes immunity, and disrupts the gut microbiota, causing high mortality rates and sub-par growth in chickens [3]. The global poultry industry incurs economic loss nearing USD 6 billion/year from NE, including the costs of production losses and those associated with control measures [4]. Additionally, the occurrence of *C. perfringens* (Cp), the causative agent of NE in poultry meat, poses a major threat to public health due to Cp being a leading foodborne pathogen [5]. Antibiotic growth promoters (AGPs) used in feed such as the antibacterial drugs virginiamycin and bacitracin have long been employed to manage NE in broilers [2]. The restricted usage of antibiotics and the rise of antibiotic-resistant strains of *C. perfringens* have escalated the need to explore alternative approaches for managing NE [3,5]. In 2013, the Food and Drug Administration (FDA) released guidelines to eliminate the use of clinically important antibiotics in animal husbandry [6]. Approaches to manage NE in the absence of AGPs have relied upon dietary and management practices. The available pool of potential substitutes for antibiotics includes organic acids, enzymes, plant extracts, prebiotics, probiotics, competitive exclusion microbial products, hen egg antibodies, bacteriophages, and vaccination [7].

A group of substances known as phytobiotic additives derived from medicinal plants and spices have demonstrated various properties such as anti-oxidative, anti-inflammatory, antimicrobial, and metabolic-modulating properties [8]. In the last two decades, phytobiotics have been examined as alternatives to AGPs in poultry for promoting growth, increasing the secretion of digestive enzymes, improving feed conversion, boosting immunity, increasing weight gain, and improving meat and egg quality [9,10,11]. As AGPs are phased out from livestock production due to the emergence of antimicrobial resistance, phytobiotics, as alternatives, are being used to improve intestinal microbiota and disease control, thereby promoting growth [11,12]. Black cumin, also known as black seed (*Nigella sativa* L.), is widely grown in countries in South Asia, Africa, and the Middle East; it is one such phytobiotic that is viable as a feed additive [13]. Several studies have assessed the antibacterial activity of black cumin, suggesting that it may serve as a potential alternative to traditional antimicrobial therapeutics [14,15,16]. Black cumin seeds have been documented to possess other biological attributes such as anti-parasitic, anti-inflammatory, anti-diabetic, anti-cancer, antioxidant, and diuretic properties [17]. However, no study has reported the investigation of the in vivo anti-*C. perfringens* (anti-Cp) activity of black cumin seeds.

Probiotics, defined as live microbial supplements, are another alternative that can be added to feed to enhance the well-being of livestock by maintaining the gut microbiota. Feeding broilers with a single microbial strain or a cocktail of different bacterial strains and/or yeasts improves the overall live performance of birds and reduces the incidence of intestinal diseases [18]. Authentic Caucasian Kefir is one such probiotic formulated by the fermentative activity of unique ‘kefir grains’ containing a wide spectrum of lactic acid bacteria, acetic acid bacteria, and yeast in a polysaccharide matrix [19]. Numerous health benefits from the consumption of true, kefir grain-derived kefir have been identified and thoroughly reviewed [19,20,21]. Cho et al. [22] reported that oral administration of milk kefir not only improved live performance in broiler chickens but also benefitted meat quality. The beneficial effect of authentic kefir is due to the symbiotic effect of a unique mixture of cultures of *Lactobacilli*, *Lactococci*, and yeast on gut health; this unique mixture of probiotic microorganisms is found only in kefir grains and products made from authentic kefir grains. However, kefir sold commercially in the United States is not authentic kefir made from kefir grains. The current study employs the use of authentic kefir prepared from kefir grains.

In our previous research, black cumin seeds oil was evaluated at levels of 1, 2, and 5 mL/kg in feed and 1:10 kefir in drinking water as effective alternatives to antibiotics in mitigating NE in chickens [16,23]. Nevertheless, the inclusion of black cumin seeds oil may incur higher costs due to the involved extraction process, and its shelf life may be shortened due to oxidation, making seeds a viable alternative. Hence, in this present study, black cumin seeds were utilized as a feed supplement along with a higher concentration of kefir in drinking water. Therefore, the purpose of this study was to evaluate *Nigella sativa* (black cumin) seeds and kefir as alternatives to antibiotics for poultry production. The successful use of black cumin seeds and kefir could improve poultry health and combat the threat of antibiotic resistance in the poultry industry.

## 2. Materials and Methods

### 2.1. Challenge Strains 

A *C. perfringens* isolate designated Cp#4, a clinical NE strain positive for alpha and NetB toxins obtained from the Southern Poultry Research Group (SPRG), Nicholson, GA, USA, was used in this study [24]. Cp#4 was cultured on brain heart infusion (BHI) agar (BD BBL, Franklin Lakes, NJ, USA) and incubated in an anaerobic environment in a Bactron EZ anaerobic chamber (Sheldon Manufacturing Inc., Cornelius, OR, USA) at 37 °C overnight.

For the broiler challenge trial, SPRG freshly prepared culture of Cp#4 and Southern Poultry Feed and Research (SPFR), Athens, GA, USA propagated *E. maxima* based on an established NE challenge model, as outlined by Hofacre et al. [25].

### 2.2. Anti-C. perfringens Activity of Black Cumin Seed Hexane Extracts

Black cumin seeds (BCS) products A, B, C, and D were procured online. BCS (3 g) powdered with a coffee grinder (Krups, Millville, NJ, USA) was extracted with hexane (9 mL) in a centrifuge tube for 1 h in a shaker at a rate of 200 rpm. The supernatant was collected by centrifuging the mixture for 10 min at 8000× *g* at ambient temperature (20 °C). After repeating the extraction procedure twice, the supernatant collected was pooled and kept at room temperature under a fume hood overnight to remove hexane by evaporation.

Anti-*C. perfringens* activity was determined by the disk diffusion method and the minimum inhibitory concentration (MIC) of BCS extracts was determined according to the Clinical and Laboratory Standards Institute methods for antimicrobial susceptibility testing of anaerobic bacteria [16,26]. In brief, for the disk diffusion assay, BCS extracts, thymoquinone (TQ), and dimethyl sulfoxide (DMSO) dried disks were positioned on Cp-inoculated Brucella blood agar plates (BD BBL, Franklin Lakes, NJ, USA) and incubated anaerobically in a Bactron EZ anaerobic chamber at 37 °C for 24 h, and the zone of inhibition (diameter) was recorded. For MIC determination, BCS hexane extracts were prepared in 2-fold serial dilutions (2, 4, 8, 16, 32, 64, and 128×) in Brucella broth supplemented with 5 μg/mL vitamin K1, 1 μg/mL hemin, and 5% sheep blood and mixed with an equal volume of Cp#4 culture and evaluated in triplicate using 96-well microplates. The plates had a final bacterial concentration of ca. 1 × 10^5^ CFU/mL per well and were incubated anaerobically in a Bactron EZ anaerobic chamber at 37 °C for 24 h. After being incubated, wells were observed for turbidity to determine the lowest concentration of BCS hexane extract that inhibited growth. The study also included a negative control (no bacterial inoculum), BCS hexane extract control (dilution without bacterial inoculum), and quality control (16 μg/mL tetracycline for *C. perfringens* strain Cp#4). The lowest concentration of BCS extract that entirely inhibited Cp growth in comparison to no treatment controls was defined as the MIC.

### 2.3. Gas Chromatography–Mass Spectrometry (GC-MS) Analysis of Black Cumin Seeds Extract

The BCS product (A), having the highest level of antimicrobial potency as determined above, underwent GC-MS analysis at the Laboratory for Environmental Analysis (University of Georgia, Athens, GA, USA). The oil extracted from crushed BCS seeds (A) was obtained using hexane, as described earlier, and subsequently dried at 60 °C in a nitrogen atmosphere. The resulting oily residue in the evaporation tubes was dissolved in 3 mL aliquots of hexane, mixed using a vortex shaker, and then transferred to 10 mL volumetric flasks. The analysis of the volatile oil utilized thymoquinone ion monitoring and was carried out on a Hewlett-Packard model 6890 gas chromatograph with similar parameters to those described by Manjunatha et al. [16]. The compounds were identified by using the Wiley MS library based on retention index and peak area percentages.

### 2.4. Kefir Culture Preparation 

Kefir was prepared using Endanem dried kefir grains (Danem Dairy and Dairy Products Ltd., Suleyman Demirel University, Isparta, Turkey). The kefir grains were activated by inoculating 2 g of dried kefir grains into 1 L of ultra high-temperature (UHT) processed whole milk (Parmalat, Lactalis American Group, Inc., Buffalo, NY, USA) and were fermented for 20 h at 25 °C to reach a final pH of 4.5–4.6 and titratable acidity (calculated as lactic acid) of 0.88–0.90 [27]. Using a sterile sieve, the kefir grains were sieved from the culture and rinsed with sterile distilled, deionized water before being transferred to fresh pasteurized milk. The kefir grains were maintained by weekly transfer into fresh milk to ensure the microbial composition (lactic acid bacteria to yeast) remained consistent. For the animal trial, the prepared kefir was transported on ice weekly to SPFR and stored under refrigeration (4 °C) until use. 

### 2.5. Screening Kefir Cultures for Anti-C. perfringens Activity using the Triple-Agar-Layer Method 

Kefir was screened to evaluate anti*-C. perfringens* activity using the triple-agar-layer method [28]. Kefir was prepared according to the procedure mentioned above, serially diluted in 0.85% saline, and then inoculated onto MRS (De Man, Rogosa, and Sharpe) (HiMedia, Mumbai, India) agar plates by spread plating. A layer of 8 mL MRS agar (1.7% *w*/*v*) supplemented with 100 mg/L nystatin was overlaid on the plates, and the plates were incubated at 30 °C. Upon 48 h of incubation, the colonies were stacked with a layer of 1% (*w*/*v*) BHI agar spiked with 10^6^ CFU/mL of Cp#4 overnight culture followed by anaerobic incubation of plates at 37 °C for 24 h in a Bactron EZ anaerobic chamber. The kefir culture colonies with inhibition zones were identified, and the numbers were recorded to calculate the percentage inhibition with respect to the initial *Lactobacilli* counts observed on MRS plates after 48 h of incubation.

### 2.6. Combined Activity of Black Cumin Seeds Hexane Extract and Kefir

To assess the combined activity of BCS hexane extract of product A and kefir, a broth growth inhibition method was carried out in which BCS hexane extract and kefir culture come in direct contact with *C. perfringens* strain Cp#4, thereby enhancing interaction between the two. BCS hexane extract and kefir culture were prepared as described above. The *C. perfringens* strain Cp#4 was grown anaerobically in a Bactron EZ anaerobic chamber on BHI agar for ~24 h, and the bacterial suspension of the colonies in 0.85% saline was modified to an optical density (OD) of ca. 0.5. The adjusted *C. perfringens* culture was inoculated into BHI broth to produce a final concentration of ca. 10^3^ CFU/mL. In addition, 0.25 mL (5%) of BCS hexane extract and 1 mL (20%) of kefir were incorporated for respective treatments to make up the final volume to 5 mL of BHI. For the positive control with 5 µL of Cp#4 culture, kefir and BCS hexane extract were replaced with an equal amount of sterile deionized water, and the negative control contained 1.25 mL of sterile deionized water and 5 µL of saline. All tubes were incubated in a Bactron EZ anaerobic chamber at 37 °C for 24 h. At 0 h and 24 h, a sample was collected from the positive and negative controls and the three treatments, which were serially diluted and plated on tryptose sulfite cycloserine (TSC) agar (HiMedia, Mumbai, India) for *C. perfringens* enumeration according to Manjunatha et al. [16].

### 2.7. Birds, Experimental Design, and Diets 

The experimental design, animal care, and management of chickens followed the National Research Council (NRC)’s guide for care and use of laboratory animals under the supervision of SPFR’s institutional animal care and use committee [29]. A total of 384 Cobb 500 (Cobb-Vantress, Cleveland, GA, USA) male broiler chicks newly hatched on day 0 (D 0) were reared in Petersime battery cages in a temperature-controlled building of SPFR with feed and water available ad libitum. The dietary composition of the basal feed and nutrient levels are shown in Table S1.

The chicks were allocated into six treatment groups in a completely randomized block experimental design, with each treatment group having eight replicates with 8 birds per replicate (Table 1). The experimental groups included three controls and three treatment groups. The controls were as follows: a negative control (no Cp#4 challenge; treatment 1), a positive control (Cp#4 challenge; treatment 2), and a bacitracin methylene disalicylate (BMD) 50 g/ton (g/t) antibiotic control (treatment 3). Treatment groups included 5% BCS (treatment 4), 20% kefir (treatment 5), and 5% BCS (D 14–28) and 20% kefir (treatment 6). All the groups received the same basal diet according to NRC’s requirements [29], with exceptions of the basal diet supplemented with BMD antibiotic (50 g/t) for antibiotic control and BCS at 5 g/kg for treatments 4 and 6. Additionally, treatment 6 received BCS only from day 14 (D 14), after coccidial challenge, until day 28 (D 28). For treatments 5 and 6, the drinking water was inoculated with kefir at a ratio of 2:10 (kefir: water, *v*/*v*) for 28 days in addition to the basal feed. 

Birds and feed were weighed on D 0, 14, 21, and 28 to evaluate live performance indicators of feed intake (FI), body weight gain (BWG), and feed conversion ratio (FCR). Cages were checked daily for mortality.

### 2.8. Necrotic Enteritis Challenge and Analysis

The challenge model consisted of birds being orally gavaged with ~5000 oocysts of *E. maxima* on D 14, and *C. perfringens* ca. 10^8^ CFU/mL (strain Cp#4) on D 19, 20, and 21.

On D 21, three birds were randomly selected from each cage, weighed, executed via cervical dislocation, and lesion-scored according to Hofacre et al. [25]. The scoring was based on a scale from 0 to 3, with scores assigned as follows: 0 for normal, 1 for slight mucus covering the small intestine, 2 for necrotic mucosa, and 3 for the most severe damage.

### 2.9. Cecal Sample Collection and Analyses 

Three chickens per cage, with three cages per treatment, were randomly selected to aseptically collect cecal contents on D 21 and D 28. The samples were stored on ice and transported to the laboratory. The pooled cecal samples for each treatment in replicates underwent processing within 24 h for microbiological analysis. In brief, 1 g of sample was weighed and mixed with 9 mL of 0.85% sterile saline. After vortexing, the samples were serially diluted for culturing Cp viable cells and Cp spores on TSC plates and total bacterial count (TBC) on tryptic soy agar (BD Difco, Franklin Lakes, NJ, USA) plates, following the methods outlined by Manjunatha et al. [16]. Additionally, the physicochemical parameters of moisture content, pH, and electrical conductivity were measured.

### 2.10. Statistical Analysis

Statistical assessment for normally distributed data was carried out using an ANOVA via JMP Pro 17 (SAS Institute, Cary, NC, USA), and significance between treatments (*p* < 0.05) was determined by Tukey’s HSD (all via a pairwise comparisons test). NE lesion scores and NE mortality data were analyzed by the non-parametric Kruskal–Wallis test as the data were not normally distributed.

## 3. Results

### 3.1. In Vitro Disk Diffusion and MIC Determination of Black Cumin Seeds Hexane Extracts

The anti-Cp activities of four commercially available black cumin seeds (BCS) products (A, B, C, and D) procured online in the US marketplace were initially assessed using the disk diffusion assay. On average, the extraction yield for all four BCS products was 30% (Table 2). Positive control of thymoquinone (TQ—3.2 μg/disk) showed a clear inhibition zone of an average 7.55 mm for Cp#4, whereas polar aprotic solvent DMSO (negative control) did not inhibit *C. perfringens*. All BCS hexane extracts showed a distinct inhibition zone against Cp#4, with BCS product A exhibiting the largest zone of inhibition of 30.75 ± 0.35 mm (*p* < 0.05) followed by products B, C, and D, respectively, and no significant difference was noticed between products C and D in terms of inhibition.

For MIC testing, 32×, 8×, 4×, and 2× dilutions were the lowest concentration of the hexane extracts of BCS products A, B, C, and D, respectively, which completely inhibited the growth of strain Cp#4. The results of MIC testing agree with the disk diffusion assay results, which indicated that product A had the highest anti-*C. perfringens* activity against strain Cp#4. Based on the results obtained from disk diffusion assay and MIC determination, product A was selected for GC-MS analysis and broiler challenge trial. 

### 3.2. GC-MS Analysis 

The major compounds identified in BCS product A by GC-MS analysis is shown in Table S2, representing 99.99% of the overall makeup of the product. P-cymene, α-terpinene, thymoquinone, carvacrol, and thymol were the major components in BCS product A with possible antimicrobial properties. In addition, other compounds of duroquinone, ocimene, terpinolene, durenol, germacrene B, citronellal, nerolidol, and ledol were also present in the product. Given thymoquinone being one of the primary antimicrobial agents labeled on BCS products, it was quantified and assessed to be 0.41 ± 0.08% in BCS product A.

### 3.3. Anti-C. perfringens Activity of Kefir Culture 

The results for screening kefir culture for anti-*C. perfringens* activity by the triple-agar-layer method revealed that after 48 h, the MRS plates had 9.06 ± 0.07 log_10_ CFU/mL *Lactobacilli* growth. When the *Lactobacilli* colonies were overlaid with BHI agar spiked with *C. perfringens* strain Cp#4 and incubated anaerobically, zones of inhibition were observed. The zones of inhibition around enumerated *C. perfringens* colonies revealed that 45.3 ± 11.6% of the kefir population inhibited the growth of Cp#4. 

### 3.4. Combined Anti-C. perfringens Activity of Black Cumin Seeds Hexane Extract and Kefir

**Figure 1:**
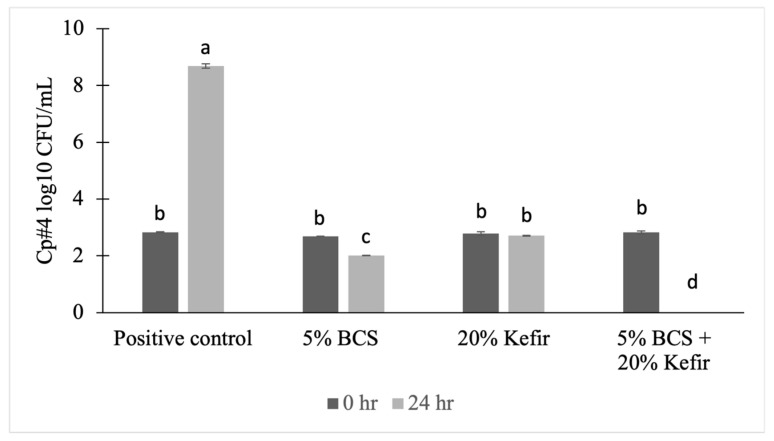
Combined activity of black cumin seeds (BCS) hexane extract and kefir against *C. perfringens* strain Cp#4. ^a, b, c, d^ Different letters indicate significant difference (*p* < 0.05) according to Tukey’s test.

The positive control of Cp#4 increased from 2.83 ± 0.02 log_10_ CFU/mL at 0 h to 8.69 ± 0.08 log_10_ CFU/mL at 24 h (Figure 1). The BCS hexane extract and kefir culture individually at 0 h had 2.69 ± 0.01 and 2.78 ± 0.07 log_10_ CFU/mL growth, respectively, and at 24 h had 2.01 ± 0.01 and 2.71 ± 0.01 log_10_ CFU/mL growth, respectively, suggesting bacteriostatic effect. The tubes for the combined treatment (5% BCS hexane extract + 20% kefir) had 2.82 ± 0.05 at 0 h and <1 log_10_ CFU/mL growth (below the detection limit) at 24 h. The results indicate that the BCS extract, kefir, and their combination significantly inhibited (*p* < 0.05) the growth of Cp#4 at 24 h, with the combination providing the best results with no growth. This indicated a bactericidal effect (below the detection limit), which may probably be attributed to the synergistic activity of BCS and kefir.

### 3.5. Live Performance of Broiler Chickens

Live performance data for the trial with regard to feed intake (FI), body weight gain (BWG), and feed conversion ratio (FCR) are presented in Table 3, Table 4 and Table 5, respectively. In terms of FI for 0 to 14 D period, the combination group (treatment 6) had the least feed intake and was similar to that of the 20% kefir group but statistically different (*p* < 0.05) from the 5% BCS group and controls (Table 3). There was no marked difference in FI noted among treatments (*p* > 0.05) except for the negative control for the 14 to 21 D period. For the 14 to 28 D period, the combination group (treatment 6) exhibited the highest feed intake, similar to the negative control, whereas the 5% BCS and the 20% kefir groups were similar (*p* > 0.05) to the positive (treatment 2) and antibiotic (treatment 3) controls. During the period spanning from 0 to 28 D, the combination group (treatment 6) had the highest feed intake except for negative control and was comparable to the 20% kefir group and positive (treatment 2) and antibiotic (treatment 3) controls; however, it was statistically distinct (*p* < 0.05) from the 5% BCS group.

Throughout the 0 to14 D interval, no notable variation (*p* > 0.05) in BWG between treatment groups and the antibiotic group was noted (Table 4). Yet, the 5% BCS treatment exhibited a significant distinction (*p* < 0.05) from the positive control (treatment 2), whereas the 20% kefir (treatment 5), combination (treatment 6) groups, and antibiotic control (treatment 3) were comparable (*p* > 0.05) to the positive control. During the 14 to 21 D period, the BWG for treatments was similar (*p* > 0.05) to that of the antibiotic control and statistically different (*p* < 0.05) from the positive control (treatment 2), excluding the 20% kefir group, which was identical to the positive control. Although the BCS and combined groups had similar BWG (*p* < 0.05) to the antibiotic control, neither of the treatments were different (*p* < 0.05) from the positive control during the 14 to 28 D period. Overall (0 to 28 D period), the BWG of all treatments was similar to that of negative and antibiotic controls and higher (*p* > 0.05) than the positive control, excluding the 20% kefir group.

The FCR during the 0 to 14 D period was the best for the combined treatment of 5% BCS and 20% kefir (treatment 6) and was comparable (*p* > 0.05) to that of the negative control (treatment 1), with the negative control not showing a substantial difference (*p* > 0.05) from the antibiotic control (treatment 3), 5% BCS, and 20% kefir treatments. The combined treatment with BCS and kefir (treatment 6) had the best FCR for the 14 to 21 D duration and was similar (*p* > 0.05) to the antibiotic control (treatment 3). Additionally, the 5% BCS group had a similar (*p* > 0.05) FCR to the antibiotic control (treatment 3). Throughout the 14 to 28 D period, all treatments had similar feed conversion and were significantly different (*p* < 0.05) from negative, positive, and antibiotic controls. Throughout the duration of 0 to 28 D, the negative control (treatment 1) and the combined group (treatment 6) exhibited the optimal FCR, and no marked difference (*p* > 0.05) between these and the antibiotic control was seen, indicating that the combined treatment with 5% BCS and 20% kefir is as effective as the antibiotic. The FCR of the antibiotic group and the 5% BCS group showed no noticeable difference (*p* > 0.05), suggesting that a 5% BCS diet is comparable in effectiveness to the antibiotic diet. Overall, for the 0 to 28 D period, identical to the antibiotic control, the combined treatment had a similar FCR to the negative control, which was notably lower (*p* < 0.05) than that of the positive control.

### 3.6. NE Score and NE Mortality Rates

The NE scores for the 5% BCS treatment and the combined 5% BCS and 20% kefir treatment were ≤0.38 (Table 6) and lower (*p* > 0.05) than the positive control (treatment 2) but surpassed the antibiotic group (treatment 3) and the negative control (treatment 1) (*p* > 0.05). However, BCS (treatment 4) and the combined groups (treatment 6) were not significantly different (*p* > 0.05) from the positive control and the antibiotic control (treatment 3). Chickens treated with 20% kefir (treatment 5) had the highest NE score of 0.63 and were substantially different (*p* < 0.05) from the antibiotic control and negative control but not from other treatments. Except for the combined treatment (treatment 6) and negative control (treatment 1), all other experimental groups had similar mortality rates (Table 6). Treatment 6 using 5% BCS and 20% kefir produced the lowest mortality of 14.1%, which was significantly lower (*p* < 0.05) than the positive control but showed no difference from the antibiotic treatment (*p* > 0.05). In summary, the combined treatment proved efficacious in lowering NE lesion scores and NE mortality compared to the positive control (treatment 2) and was similar to the antibiotic treatment (*p* > 0.05).

### 3.7. Cecal Sample Analysis 

Table S3 details the moisture content, pH, and electrical conductivity of cecal contents collected on D 21 and 28. Cecal contents exhibited a moisture content varying between 77.7 and 81.1%, pH between 6.70 and 7.02, and electrical conductivity ranging from 2.66 to 3.22 ms/cm. These results displayed no noticeable pattern, suggesting that neither BCS, kefir, nor their combination had a significant impact on the physical characteristics of cecal samples. 

The results of the microbial enumeration of Cp viable cells, Cp spores, and the total bacterial count of cecal samples collected on D 21 and 28 are reported in Table S4. Total bacterial counts among the controls and treatments for cecal samples fell within the range of 9.07–9.68 log_10_ CFU/g, with no discernible pattern identified. On D 21, Cp vegetative cells were lower in all treatments, excluding the positive control (treatment 2), and were higher than the negative control (treatment 1); the combined group (treatment 6) was similar (*p* > 0.05) to that of the antibiotic treatment (treatment 3). Cp spores on D 21 for kefir (treatment 5) and the combination of 5% BCS and 20% kefir (treatment 6) were markedly lower (*p* < 0.05) compared to the positive control but higher than that of the antibiotic control (treatment 3), but the BCS treated group was no different (*p* > 0.05) from the positive control. At 28 D, Cp vegetative cells were lower (*p* < 0.05) for treatments 5 and 6 than the positive and antibiotic controls. There was no statistically noticeable distinction between the negative control (treatment 1) and treatment 6 for Cp vegetative cells on D 28. In addition, there was no notable difference on D 28 between controls and treatments in terms of spores, excluding the BCS-treated group. Except for Cp spores on D 21 and both Cp vegetative cells and spores on D 28 associated with BCS treatment, nearly all treatments showed a significant reduction (*p* < 0.05) in Cp vegetative cells and spores on D 21 and D 28 compared to the positive control. To summarize, during the period from D 21 to D 28, there was a reduction observed in both the vegetative cells and spores of Cp across all treatments, suggesting a decline in Cp colonization over time.

## 4. Discussion

While some research has explored the benefits of phytobiotics of *Nigella sativa* seeds in enhancing the live performance of broilers, there has been no investigation into the impact of supplementing feed with BCS specifically for NE challenged broilers. In animal studies published previously, a basal diet augmented with BCS was evaluated in the 0.5 to 5% range for dietary and antimicrobial assessment objectives [30,31,32]. The current investigation examined a basal diet supplemented with 5% BCS. BCS product A demonstrated the most potent antibacterial effectiveness against Cp#4 in vitro, leading it to be selected for the animal trial.

A previously published compositional analysis of black cumin seeds revealed components of 20.85% protein, 38.20% fat, 4.64% moisture, 4.37% ash, 7.94% crude fiber, and 31.94% total carbohydrates [33]. Various other pharmacologically active compounds have also been identified, including 30–48% of thymoquinone (TQ), thymol, thymohydroquinone, dithymoquinone, 7–15% of p-cymene, 6–12% of carvacrol, 1–8% of sesquiterpene longifolene, 2–7% of 4-terpineol, 1–4% of t-anethol, and α-pinene [17,34]. The findings of the present study revealed the existence of constituents such as p-cymene, thymol, thymoquinone, and carvacrol in BCS product A, consistent with findings from the studies mentioned above.

In the present study, thymoquinone, a major antimicrobial compound in BCS, was quantified and determined to be 0.41 ± 0.08%, which is similar to the results of Işık et al. [35], who documented 0.01–0.38% TQ. The difference in the chemical compositions of *N. sativa* seeds may arise from differences in extraction techniques, cultivars, and plant origins [36]. Owing to the qualitative aspect of the method, additional quantifiable exploration is required for accurately identifying and determining the concentration of compounds.

Kefir, a probiotic fermented milk product, is traditionally crafted through milk fermentation by a symbiotic balance of *Lactobacillus* (*L. lactis*, *L. helveticus*, *L. casei*), *Streptococcus* (*S. cremoris, S. lactis*), and yeasts found in kefir grains [19]. Kefir grains consisting of specific species of bacteria and yeasts are crucial for authentic kefir production. Conversely, commercial kefir available in the United States often lacks the diversity of beneficial and characteristic bacteria, yeasts, and kefiran present in genuine kefir. Authentic milk kefir products comprise proteins, fat, polysaccharides, minerals, vitamins, and lactic acid with beneficial probiotic and prebiotic components [37]. Studies like those of Thoreux and Schmucker [38] and Cenesiz Özcan [39] highlight the positive impact of milk kefir on immune responses in rats and the performance of broiler chicks. Additionally, authentic kefir shows inhibitory properties against both Gram-negative and Gram-positive bacterial pathogens commonly present in food [40,41]. The anti-*C. perfringens* activity results of authentic kefir assessed in this study demonstrated effectiveness against the Gram-positive bacteria Cp. The unique microbiota introduced during fermentation with kefir grains contributes to these potent antimicrobial effects, distinguishing it from commercially produced kefir [19]. Despite research primarily focusing on kefir’s growth-promoting effects, its potential for disease prevention in broiler chicks remains understudied.

The coccidial infection is regarded as the most significant predisposing factor of NE caused by Cp and, hence, *Eimeria* spp. oocysts were administered in the present animal trial on D 14. The signs of depressed birds, ruffled feathers, intestinal lesions, and NE-related fatality seen in birds proves that NE was successfully induced in the broilers. The reduction in BWG and impaired FCR in the positive control could be attributed to the intestinal impairment caused by *Eimeria* and Cp challenge as the absorption and uptake of nutrients was negatively impacted. Intestinal damage correlates with reduced feed intake, decreased feed conversion, and impaired growth [42,43]. In the current study, enhanced FI, BWG, and FCR with antibiotic BMD administration suggests that *Eimeria* and Cp were suppressed by the antibiotics, thereby having a positive impact on performance factors.

A search of the literature did not reveal any studies evaluating the impact of black cumin seeds, kefir, and their combination as alternatives to antibiotics in broiler chickens challenged with Cp. The current broiler challenge study inoculated with Cp demonstrated that supplementation of BCS as a pyhtobiotic, kefir as a probiotic, and their combination improved the BWG and FCR of broilers. Combination treatment with 5% BCS and 20% kefir showed the best FCR, followed by 5% BCS treatment, and was similar (*p* > 0.05) to that of the antibiotic control. This may be attributed to the higher BWG observed in the combined treatment, which could be due to the occurrence of bioactive constituents of thymoquinone, carvone, carvacrol, and terpineol in BCS and the impact of authentic milk kefir on mucosal immune response. Previous research has reported that BCS constituents possess antimicrobial activity against parasites and pathogenic bacteria, leading to better feed intake [31,44]. Toghyani et al. [45] documented that supplementation of 2% milk kefir in broilers enhanced the body weight at 28 and 42 days (*p* < 0.05). In another study by Guler et al. [30], supplementing the diet with ground BCS at 1% (10 g/kg) improved BWG and FCR. In the current study, broiler performance showed a positive impact after dietary administration of 5% BCS and 20% kefir. 

In terms of NE score, BCS and the combined treatment reduced lesion scores and were comparable with the antibiotic group. All the treatment groups showed reduced NE mortality and were similar (*p* > 0.05) to the antibiotic control. Live performance, NE score, and NE mortality indicate that the combination treatment was the most effective, followed by 5% BCS and 20% kefir cultures, both having similar results. During the animal trial, for treatment 6, feed was supplemented with BCS during D 14–28, and the kefir culture was provided in drinking water throughout the 28 D period. The reasoning behind this strategy was that kefir can build immunity in chickens by improving intestinal microbiota; this was followed by administration of BCS after disease inoculation, counteracting the *C. perfringens* post coccidial infection. In the current study, the synergistic activity results (Figure 1) of BCS and kefir corroborate the animal trial findings.

Probiotics such as in authentic kefir decrease the occurrence of intestinal illnesses and improve the overall live performance of chickens [46]. Probiotics mitigate the chance of subclinical NE by producing antimicrobial substances that inhibit proliferation of pathogenic bacteria, thereby improving host immune response, maintaining the intestinal microflora equilibrium, and boosting metabolism [47]. Probiotics also exhibit competitive exclusion by competing for nutrients with bacterial pathogens in the intestine of chickens [48]. Therefore, the probable mechanism of action of probiotics includes competitive exclusion, improving digestive enzymatic activity, production of counteractive toxins and compounds that can inhibit pathogen growth, modulation of the host’s immune system, and alteration of the gut microbial balance [49]. The authentic kefir used in this study contains more than 30 different probiotic microorganisms, including lactic acid bacteria such as *L. acidophilus*, *L. helveticus*, *S. thermophilus*, *B. bifidum*, *L. kefiranofaciens*, and *L. kefiri*, and yeasts such as *K. marxianus*. These microorganisms have been identified in traditional kefir prepared from authentic kefir grains, and these starter cultures have been associated with antimicrobial and anti-inflammatory properties [50].

There were no discernible patterns (*p* > 0.05) seen for the total bacterial counts across all treatments and controls. All the treatments under investigation significantly lowered (*p* < 0.05) Cp vegetative cells and spore counts during challenge, substantiating the anti-*C. perfringens* activities of BCS and kefir.

Synergistic feed additives are believed to combine individual beneficial effects, leading to a superior effect compared to the benefits of individual applications [51]. The same principle holds true for combinations of probiotics and prebiotics, with the intention of encouraging beneficial bacterial growth while providing substrates for probiotics [52]. Previous research has shown that probiotics do not diminish the overall quantity or activity of gut bacteria; however, they elevate concentrations of beneficial metabolites in broiler chickens [53,54]. On the other hand, phytobiotics find application due to their antibacterial activity [55]. Additional investigations on combinations of feed additives like organic acids mixed with phytases [56], probiotics [57], or phytobiotics [58] have been reported. To our understanding, no study has evaluated the use of both phytobiotic and probiotics for disease prevention in poultry production. The current investigation is believed to be the first to examine the impact of these additives in combination on alleviating NE. The phytobiotic action of BCS and the probiotic action of kefir are each known to affect the poultry intestinal microbiota [59]. Future research should include studying the mechanisms of possible synergistic effects of BCS and kefir with a comprehensive analysis of the intestinal microbiome using high-throughput metagenomic analyses.

Although an economic evaluation of BCS and kefir was not conducted in the study, it is important to consider their cost-effectiveness as proposed additives. BCS supplementation is more economical than its essential oil due to the extraction process involved. Research has shown that that incorporating BCS into basal diets reduces production costs and increases profitability. Khadr and Abdel-Fattah et al. (2006) reported that a diet fortified with 2% black seeds improved performance without increasing the overall diet cost [60]. Ahmad (2005) and Ihsan (2003) found that broilers fed with BCS meal were more profitable compared to those without the supplement [61,62]. Further, in the current research, kefir (in wet form) was produced from reusable kefir grains, eliminating the need for freeze-dried starter culture. Economically, the cost of producing kefir in its wet form ($1.7/kg) is 3.5 times cheaper than its freeze-dried form ($5.8/kg) [63,64]. The primary cost factor in kefir production is investment in freeze-drying machinery, accounting for 57% of the total investment. Additionally, the cost of the kefir treatment was significantly reduced as kefir was diluted with drinking water in this study.

Numerous studies suggest that phytobiotics or probiotics could be incorporated as AGPs substitutes in feed. However, the effectiveness of these alternatives relies on factors such as concentration of uptake, whole diet, method of supplementation, and poultry environment [65]. Efficiency of alternate feed additives could be enhanced by applying a combination of phytobiotics and probiotics in accordance with synergistic activity, which could yield a more favorable solution than a single additive. The current study examined the synergistic effects of BCS phytobiotics and kefir probiotics as alternatives to commercially used bacitracin antibiotics and justified synergism as a favorable solution.

One limitation of this study was the use of whole black cumin seeds, which resulted in reduced bioavailability of bioactive nutrients. Some intact black cumin seeds were observed in the gizzard and feces of the chickens, indicating inability of the chickens to fully digest the whole seeds. Future studies should include freshly ground black cumin seeds to investigate the improved bioavailability of phytobiotic components. Another limitation was the lack of efficacy of culture-based methods in distinguishing background Cp from the inoculated *C. perfringens* in cecal samples, primarily due to the common occurrence of *C. perfringens* in chicken gut and the environment. To delve deeper into determining the source of *C. perfringens* in birds, further exploration using a real-time PCR assay will be necessary.

## 5. Conclusions

The findings of this study disclosed that 5% BCS, 20% kefir, and their combination effectively reduced NE lesion scores and mortality without any negative effects on broiler live performance compared with infected positive control. The 5% BCS, 20% kefir, and combination treatments were as effective as the antibiotic treatment in decreasing mortality during NE infection, suggesting *Nigella sativa* (black cumin) and authentic kefir have the potential to serve as alternatives to frequently administered antibiotics such as BMD in order to improve broiler performance, particularly when the birds are susceptible to necrotic enteritis. The combination of kefir and BCS exhibits potential synergistic effects, proving to be the most effective remedy for disease control. Hence, the successful utilization of *Nigella sativa* and authentic kefir can contribute to enhancing poultry health and consequently addressing the challenge of antibiotic resistance in the poultry industry.

## 6. Intellectual Property Development

The kefir cultures used in this project are the proprietary assets of Danem Dairy and Dairy Products Ltd., Suleyman Demirel University, Isparta, Turkey. 

## Figures and Tables

**Table 1:** Treatments and treatment schedule for animal trial.

Treatments	*E. maxima*	*C. perfringens* Strain Cp#4	Cages/Trt
1. Negative control	D * 14	No	8
2. Positive control	D 14	D 19, 20, 21	8
3. Antibiotic control (BMD 50 g/t)	D 14	D 19, 20, 21	8
4. 5% Black cumin seeds (BCS)	D 14	D 19, 20, 21	8
5. 20% Kefir	D 14	D 19, 20, 21	8
6. 5% BCS (D 14–28) and 20% kefir	D 14	D 19, 20, 21	8

* D refers to the day of the trial.

**Table 2:** Percentage yield of oil and anti-*C. perfringens* activity of commercially available black cumin seeds (BCS) products.

BCS Products	Percentage Yield of Oil	Inhibition Zone (mm) for BCS Hexane Extract Tested against Cp#4
A	30.25 ± 0.01 ^1a^	30.75 ± 0.35 ^1a^
B	30.83 ± 0.0 ^a^	18.25 ± 0.35 ^b^
C	29.42 ± 0.01 ^a^	15.25 ± 0.35 ^c^
D	29.67 ± 0.02 ^a^	15.00 ± 0.00 ^c^

^1^ Data are presented as mean ± S.D. as the average of three replicates, with TQ (3.2 µg/disk concentration) as a positive control (zone of 7.55 ± 0.11 mm against Cp#4) and DMSO as a negative control. ^a, b, c^ Different letters in the same column indicate significant difference (*p* < 0.05) according to Tukey’s test.

**Table 3:** Effect of supplementation of black cumin seeds (BCS) and kefir on feed intake (FI).

Treatment	Total Cage Feed Intake (FI) (kg)			
	D * 0–14	D 14–21	D 14–28	D 0–28
1. Negative control	7.22 ± 0.50 ^1a^	3.89 ± 0.31 ^a^	7.32 ± 0.50 ^a^	10.65 ± 0.31 ^a^
2. Positive control	6.82 ± 0.86 ^ab^	3.54 ± 0.36 ^ab^	5.91 ± 0.43 ^b^	9.19 ± 0.88 ^bc^
3. Antibiotic control (BMD 50 g/t)	6.58 ± 0.46 ^b^	3.60 ± 0.34 ^ab^	5.67 ± 1.29 ^b^	8.64 ± 1.26 ^bc^
4. 5% BCS	6.72 ± 0.49 ^ab^	3.65 ± 0.28 ^ab^	5.22 ± 0.63 ^b^	8.29 ± 0.73 ^c^
5. 20% Kefir	6.46 ± 0.58 ^bc^	3.44 ± 0.34 ^b^	5.74 ± 1.04 ^b^	8.76 ± 1.17 ^bc^
6. 5% BCS (D 14–28) and 20% kefir	5.87 ± 0.73 ^c^	3.49 ± 0.50 ^b^	7.05 ± 0.92 ^a^	9.42 ± 0.70 ^b^

* D refers to the day of the trial. ^1^ Data are presented as mean ± S.D for 8 cages. ^a, b, c^ Different letters in the same column indicate significant difference (*p* < 0.05) according to Tukey’s test.

**Table 4:** Effect of supplementation of black cumin seeds (BCS) and kefir on body weight gain (BWG).

Treatment	Body Weight Gain (BWG) (kg/Bird)			
	D * 0–14	D 14–21	D 14–28	D 0–28
1. Negative control	0.57 ± 0.06 ^1a^	0.34 ± 0.03 ^a^	0.81 ± 0.06 ^a^	1.04 ± 0.08 ^a^
2. Positive control	0.45 ± 0.04 ^c^	0.24 ± 0.02 ^d^	0.63 ± 0.06 ^b^	0.85 ± 0.06 ^b^
3. Antibiotic control (BMD 50 g/t)	0.50 ± 0.05 ^bc^	0.27 ± 0.04 ^bc^	0.78 ± 0.19 ^a^	1.00 ± 0.19 ^a^
4. 5% BCS	0.51 ± 0.04 ^b^	0.28 ± 0.02 ^b^	0.73 ± 0.19 ^ab^	0.97 ± 0.20 ^ab^
5. 20% Kefir	0.47 ± 0.04 ^bc^	0.24 ± 0.03 ^cd^	0.63 ± 0.11 ^b^	0.86 ± 0.12 ^b^
6. 5% BCS (D 14–28) and 20% kefir	0.51 ± 0.09 ^bc^	0.28 ± 0.04 ^b^	0.73 ± 0.07 ^ab^	0.96 ± 0.04 ^ab^

* D refers to the day of the trial. ^1^ Data are presented as mean ± S.D. ^a, b, c, d^ Different letters in the same column indicate significant difference (*p* < 0.05) according to Tukey’s test.

**Table 5:** Effect of supplementation of black cumin seeds (BCS) and kefir on feed conversion ratio (FCR).

Treatment	Feed Conversion Ratio (FCR = FI:BWG)			
	D * 0–14	D 14–21	D 14–28	D 0–28
1. Negative control	1.58 ± 0.10 b ^1bc^	1.42 ± 0.04 ^e^	1.43 ± 0.05 ^d^	1.53 ± 0.08 ^d^
2. Positive control	1.88 ± 0.19 ^a^	1.86 ± 0.10 ^a^	1.98 ± 0.11 ^a^	1.95 ± 0.13 ^a^
3. Antibiotic control (BMD 50 g/t)	1.66 ± 0.11 ^b^	1.66 ± 0.11 ^cd^	1.58 ± 0.08 ^c^	1.61 ± 0.07 ^cd^
4. 5% BCS	1.67 ± 0.09 ^b^	1.70 ± 0.05 ^bc^	1.75 ± 0.09 ^b^	1.70 ± 0.08 ^bc^
5. 20% Kefir	1.72 ± 0.08 ^b^	1.77 ± 0.14 ^ab^	1.78 ± 0.08 ^b^	1.74 ± 0.08 ^b^
6. 5% BCS (D 14–28) and 20% kefir	1.48 ± 0.22 ^c^	1.59 ± 0.11 ^d^	1.70 ± 0.04 ^b^	1.58 ± 0.10 ^d^

* D refers to the day of the trial. ^1^ Data are presented as mean ± S.D. ^a, b, c, d, e^ Different letters in the same column indicate significant difference (*p* < 0.05) according to Tukey’s test.

**Table 6:** Effect of supplementation of black cumin seeds (BCS) and kefir on necrotic enteritis (NE) lesion scores and mortality in broiler chickens.

Treatment	NE Score (0–3)	NE Mortality	
		Number	%
1. Negative control	0.0 ^c^	0/64	0.0 ^c^
2. Positive control	0.50 ^ab^	20/64	31.25 ^a^
3. Antibiotic control (BMD 50 g/t)	0.21 ^bc^	15/64	23.44 ^ab^
4. 5% BCS	0.38 ^abc^	20/64	31.25 ^a^
5. 20% Kefir	0.63 ^a^	15/64	23.44 ^ab^
6. 5% BCS (D * 14–28) and 20% kefir	0.29 ^abc^	9/64	14.06 ^b^

* D refers to the day of the trial. ^a, b, c^ Different letters in the same column indicate significant difference (*p* < 0.05) according to the Kruskal–Wallis test.

## Data Availability

The authors confirm that the data supporting the findings of this study are available within the article and in Supplementary Tables S1–S4.

## Supplementary Materials

The following supporting information can be downloaded at: https://www.mdpi.com/article/10.3390/ani14142074/s1. Table S1: Composition and nutrient contents of basal diet and black cumin seeds (BCS)-supplemented diet; Table S2: Chemical composition of black cumin seeds product A analyzed by GC-MS; Table S3: Effect of supplementation with black cumin seeds (BCS) and kefir against strain Cp#4 on physical parameters of cecal samples in broiler chickens; Table S4: Microbiological analysis of cecal samples of broiler chickens.
