# Supplementary material for: Combined Effect of Nigella sativa and Kefir on the Live Performance and Health of Broiler Chickens Affected by Necrotic Enteritis
Source: Animals (Basel). 2024 Jul 15;14(14):2074. doi: 10.3390/ani14142074 (PMC11273500; doi:10.3390/ani14142074)
Supplement: Supplementary file 1 [file animals-14-02074-s001.zip › animals-3083426-supplementary.pdf]

Supplementary Materials

Table S1. Composition and nutrient contents of basal diet and black cumin seeds (BCS) supplemented diet

Ingredient	Composition (%)	
	Basal feed (Treatments 1, 2, and 5)	Black cumin seed (5%) (Treatments 4 and 6)
Corn, yellow, grain	53.17	48.80
Soybean meal dehulled, solvent	40.18	38.82
Vegetable fat	2.42	3.08
Dicalcium phosphate	2.03	2.07
Calcium carbonate	1.21	1.17
Salt (NaCl)	0.38	0.39
Methionine MHA	0.38	0.41
L-lysine	0.09	0.14
Trace mineral premix[1]	0.08	0.08
Vitamin premix[2]	0.07	0.07
Calculated nutrients		
Crude protein	22.08	22.34
Metabolizable energy, kcal/kg	3,008.00	3,008.00
Digestible methionine	0.61	0.62
Digestible lysine	1.10	1.10

[1]Trace mineral mix provided the following (per kg of diet): 60 mg of manganese ($MnSO_4 \cdot H_2O$); 30 mg of iron ($FeSO_4 \cdot 7H_2O$); 50 mg of zinc (ZnO); 5 mg of copper ($CuSO_4 \cdot 5H_2O$); 0.15 mg of iodine (ethylene diamine dihydroiodide); 0.3 mg of selenium ($NaSeO_3$). [2]Vitamin mix provided the following (per kg of diet): 2.4 mg of thiamine mononitrate; 44 mg of nicotinic acid; 4.4 mg of riboflavin; 12 mg of D-Ca pantothenate; 12.0 μg of vitamin B_{12} (cobalamin); 4.7 mg of pyridoxine·HCl; 0.11 mg of D-biotin; 5.5 mg of folic acid; 3.34 mg of menadione sodium bisulfite complex; 220 mg of choline chloride; 27.5 μg of cholecalciferol; 6,306.6 IU of *trans*-retinyl acetate; 11 IU of all-*rac* α-tocopheryl acetate; 125 mg of ethoxyquin.

Table S2: Chemical composition of black cumin seed product A analyzed by GC-MS

RI[1] (min)	Area %[2]	Compounds Identified
5.56±0.39[3]	0.47	p-cymene, m-cymene, m-xylene, m-di-tert-butylbenzene, p-di-tert-butylbenzene, N-phenylbutanimide
7.04±0.27	50.78	thymoquinone, duroquinone, α-pinene, α-terpinene, isoterpinolene, 2-carene, α-fenchene, sabinene, γ-elemene, δ-eiemene, germacrene b, camphene, allo-ocimene, isopseudocumenol, dimethyloctatriene, 2,3-dimethylanisole, benzene, benzyl alcohol, 1,5,5-trimethyl-6-methylene-cyclohexene
6.20±0.09	5.87	thymoquinone, duroquinone, ocimene, lumazine, α-terpinene, terpinolene, 2,6-dimethylanisole, isopseudocumenol, 2,5-dimethylanisole
11.18±0.65	21.7	(+)-valencene, 5-decene, cyclodecene, 2-hydroxy-6-methoxyacetophenone, 1-(2,4-dihydroxy-3-methylphenyl) ethanone, p-tert-butylcatechol, cis-δ4-tetrahydrophthalimide, 5-nitro-m-xylene, methyl carbanilate, [s-(e,e)]-3,8-dimethyldeca-4,6-diene, 2-chloro-2-methylpentane, hexadecylene oxide
7.40±0.53	2.11	carvacrol, thymol, durenol, germacrene B, γ-elemene, δ-eiemene, β-bisabolene, bicyclogermacrene, 6-ethyl-3,4-dimethylphenol, 2,5-diethylphenol, 3,4-diethylphenol, 2,3,4,6-tetramethylphenol, 3,5-di-tert-butylphenol
12.83±1.48	79.64	δ-fenchane, linoleic acid, 9-eicosyne, 9-tetradecenol, Z, 1-penatdecyne, 1-heptadecyne, 9-octadecyne, methyl linolelaidate, ethyl linoleate, 11,14-eicosadienoic acid, methyl ester, 1,12-tridecadiene, 3,4-octadiene, 7-methyl-, cis-7-dodecen-1-ol

[1]The retention index (RI) is average of RI of similarly identified compounds. [2]The area percentages (Area %) are the sums of Area% of similarly identified compounds. [3]Data are presented as mean±S.D. of similarly identified compounds.

Table S3. Effect of supplementation of black cumin seeds (BCS) and kefir on physical parameters of cecal samples in broiler chickens

Treatment	D* 21			D 28		
	Moisture content (%)	pH	Electrical conductivity (ms/cm)	Moisture content (%)	pH	Electrical conductivity (ms/cm)
1. Negative control	79.19±0.08[1bc]	6.80±0.03[bc]	3.00±0.05[a]	79.67±0.13[a]	6.95±0.09[ab]	3.22±0.11[a]
2. Positive control	79.51±0.01[b]	6.70±0.02[c]	2.90±0.02[ab]	80.16±0.12[a]	7.02±0.00[a]	3.04±0.01[ab]
3. Antibiotic control (BMD 50 g/t)	81.09±0.01[a]	6.86±0.04[ab]	2.76±0.06[bc]	79.85±0.08[a]	6.95±0.04[a]	2.96±0.04[ab]
4. 5% BCS	78.99±0.18[c]	6.72±0.00[c]	2.81±0.00[abc]	79.39±0.06[a]	6.79±0.01[bc]	2.80±0.12[b]
5. 20% Kefir	78.51±0.02[d]	6.89±0.00[a]	2.94±0.11[ab]	77.69±0.82[b]	6.78±0.02[c]	2.92±0.09[ab]
6. 5% BCS (D 14-28) and 20% kefir	79.15±0.06[c]	6.72±0.02[c]	2.66±0.02[c]	80.32±0.20[a]	6.98±0.01[a]	2.85±0.05[b]

*D refers to the day of trial. [1]Data are represented as mean±S.D. of 2 replicates. [a,b,c,d]Different letters in the same column indicate significant difference ($P<0.05$), Tukey’s test.

Table S4. Microbiological analysis of cecal samples of broiler chickens

Treatment	Plate count (log_{10} CFU/g) at D[*] 21			Plate count (log_{10} CFU/g) at D 28		
	TBC[1]	Cp[2]	Cp spores	TBC	Cp	Cp spores
1. Negative control	9.52±0.02[3ab]	5.46±0.01[e]	5.45±0.01[c]	9.47±0.02[ab]	4.05±0.12[cd]	3.76±0.07[b]
2. Positive control	9.63±0.03[a]	8.03±0.04[a]	6.44±0.01[a]	9.27±0.21[ab]	5.23±0.06[b]	4.15±0.06[b]
3. Antibiotic control (BMD 50 g/t)	9.52±0.06[ab]	6.85±0.01[d]	4.91±0.03[d]	9.68±0.03[a]	4.23±0.08[c]	4.30±0.08[b]
4. BCS 5%	9.53±0[ab]	7.85±0.02[b]	6.59±0.03[a]	9.47±0.08[ab]	5.73±0.04[a]	5.37±0.01[a]
5. Kefir 20%	9.44±0.01[bc]	7.60±0.02[c]	5.67±0.02[b]	9.07±0.25[b]	2.74±0.11[e]	3.46±0.60[b]
6. BCS 5% (D 14-28) and kefir 20%	9.32±0.03[c]	6.93±0.06[d]	5.32±0.09[c]	9.36±0.12[ab]	3.79±0.12[d]	4.06±0.21[b]

[*]D refers to the day of trial. [1]TBC -Total bacterial count. [2]Cp – *C. perfringens*. [3]Data are represented as mean±S.D. of 2 replicates. [a,b,c,d,e]Different letters in the same column indicate significant difference ($P<0.05$), Tukey's test.
